# Organ size in small infants (The OSSI Study): are sonographic reference intervals applicable in preterm infants small for gestational age?

**DOI:** 10.1007/s00431-026-07580-4

**Published:** 2026-10-28

**Authors:** Lara Klischke, Diane Miriam Renz, Thomas Müller, Lars Brodowski, Doris Franke, Alexandros Rahn

**Affiliations:** 1 Department of Pediatric Pulmonology, Allergology and Neonatology, Hannover Medical School, Carl-Neuberg-Str. 1, 30625 Hannover, Germany; 2 Department of Pediatric Radiology, Institute of Diagnostic and Interventional Radiology, Hannover Medical School, Carl-Neuberg-Str. 1, 30625 Hannover, Germany; 3 Department of Obstetrics and Gynecology, Hannover Medical School, Carl-Neuberg-Str. 1, 30625 Hannover, Germany; 4 Department of Pediatric Kidney, Liver and Metabolic Diseases, Hannover Medical School, Carl-Neuberg-Str. 1, 30625 Hannover, Germany

**Keywords:** Small for gestational age, Preterm infants, Reference intervals, Abdominal ultrasound, Organ size

## Abstract

Sonographic reference intervals for liver, spleen, and kidney size in preterm infants below 40 cm body length were recently established in an appropriately grown (AGA) neonatal cohort. Whether these intervals apply to infants born small for gestational age (SGA) remains unexamined. In this cross-sectional study, 20 preterm SGA infants below 40 cm body length (mean gestational age 29 ^1^/_7_ weeks, median birth weight 695 g) underwent standardized abdominal ultrasound, measuring craniocaudal liver length in three planes, splenic length, and renal volume. Measurements were compared with published reference intervals, and z-scores assessed systematic deviation. Weight and length at examination were comparable between the SGA and AGA cohorts. The mean z-score was significantly below zero only for liver length in the midclavicular line (− 0.79, *p* = 0.014). The proportion of infants outside the reference interval exceeded the expected 5% for liver length in the midclavicular line (25%, *p* = 0.003) and anterior axillary line (20%, *p* = 0.016) and for spleen length (20%, *p* = 0.016), but rested on 4 to 5 infants each, and several values lay within 1 mm of the limit. Kidney measurements did not differ from the reference. Organ size correlated most strongly with body weight, and the deviations persisted at equal weight (post hoc).

*Conclusion*: Abdominal organ dimensions in SGA preterm infants frequently fall outside AGA-derived reference intervals, particularly for liver and spleen size, suggesting that these intervals cannot be reliably applied to this population. Prospective studies establishing reference values for SGA infants are warranted.

**Table:** 

**What is Known:** *• Sonographic reference intervals for abdominal organ size in preterm infants below 40 cm exist only for appropriately grown (AGA) infants; their applicability to infants small for gestational age (SGA) has not been examined*.	
**What is New:** *• In 20 preterm SGA infants, the mean z-score for liver length (midclavicular line) was below the AGA reference, and liver and spleen lengths fell outside the intervals more often than the expected 5% (exploratory)*. *• AGA intervals should not be assumed to apply to SGA infants*.

**Supplementary Information:**

The online version contains supplementary material available at https://doi.org/10.1007/s00431-026-07580-4.

## Introduction

Small for gestational age (SGA) is defined as a birth weight below the 10th percentile for gestational age and includes constitutionally small infants; it is not identical to fetal growth restriction (FGR). Among very preterm infants born before 32 weeks across 11 European countries, the prevalence of SGA ranged from 31.8% to 34.0%, depending on the reference standard [1]. SGA status is associated with increased mortality and major morbidity in preterm infants [2], with morbidity risk increasing with the severity of growth restriction [3]. The causes of SGA are heterogeneous and include placental, maternal, fetal, and genetic factors. Placental insufficiency, the predominant cause of FGR [4], may result in asymmetric intrauterine growth restriction, with relative preservation of cerebral growth through redistribution of fetal blood flow toward the brain in response to hypoxia („brain-sparing“-effect). This adaptive response may affect the growth of peripheral organs, including the liver, kidneys, and spleen [5, 6]. Whether these differences in organ growth persist after birth remains unclear.

Postnatal differences in renal size have been reported in preterm SGA infants: in a case–control study including 20 preterm SGA infants, 20 preterm appropriate-for-gestational-age (AGA) infants, and 20 full-term infants, renal length and transverse diameter differed significantly in the SGA group by day seven of life [7]. Comparable postnatal data on liver and spleen dimensions in preterm SGA infants could not be identified.

Accurate assessment of organ size is important to distinguish normal variation from organ hypoplasia, enlargement, or other structural abnormalities. For very and extremely preterm infants with a body length below 40 cm, sonographic reference intervals (RI) for liver, spleen, and kidney size have only recently been established in an AGA cohort, in which organ size correlated most closely with body weight [8].

If SGA is associated with altered postnatal organ dimensions, applying RIs derived from AGA infants could lead to misinterpretation of organ size. This may be particularly relevant when distinguishing reduced organ size related to fetal growth restriction from pathological organ hypoplasia.

No sonographic reference intervals exist for preterm SGA infants below 40 cm body length, and whether the AGA RIs apply to them has not been investigated. We therefore examined whether abdominal organ dimensions in SGA infants fall within the previously established AGA RIs for liver, spleen, and kidney size.

## Methods

### Patients and setting

In this cross-sectional study, preterm infants with a birth weight below the 10th percentile for gestational age according to German reference percentiles for singleton neonates [9] and a body length below 40 cm at the time of the first ultrasound examination were consecutively included in the study. No limit was set for gestational or postnatal age. Recruitment ran from April 2024 through August 2025 at a single level III neonatal intensive care unit (NICU). Weight was obtained undressed on a calibrated scale; body length and head circumference were measured with the infant supine using a flexible tape. Conditions that could affect organ size led to exclusion: systemic infection requiring intravenous antibiotics (clinical and laboratory findings); hepatic disease (direct bilirubin > 1 mg/dL, transaminases above age-adjusted limits, structural or vascular liver abnormalities on ultrasound, or metabolic liver disease on newborn screening); focal organ lesions; major abdominal wall or visceral malformations; and significant congenital heart disease. Infants who died or were transferred to an external hospital before ultrasound or whose guardians did not consent were also excluded. An isolated structural renal anomaly (e.g., duplex kidney or horseshoe kidney) did not lead to exclusion of the infant, only the affected kidney volume was excluded. The study was conducted in accordance with the Declaration of Helsinki. Ethics approval was granted by the local ethics committee (No. 11351_BO_K_2024), and legal guardians provided written informed consent before any examination.

### Ultrasound examination

Scans were acquired with a GE Venue Go™ R4 system (GE Healthcare Technologies, Chicago, IL, USA) equipped with an 8 C micro-convex probe (4—10 MHz), chosen to visualize the abdominal organs across the whole range of body sizes. Infants remained supine and unsedated and were scanned shortly before a scheduled feed to limit feeding related variability. All examinations were carried out by one of two DEGUM level II certified pediatric sonographers (German Society for Ultrasound in Medicine; 6 and 9.5 years of experience), applying a shared protocol based on DEGUM recommendations and adapted to common pediatric ultrasound practice [10, 11]; the interobserver reproducibility of this protocol has been established in a prior validation study (intraclass correlation coefficient (ICC): 0.929—0.964) [12]. Three anatomical planes were used to measure craniocaudal liver length: the midsternal line (MSL), using the aorta as the anatomical landmark; the midclavicular line (MCL), typically intersecting the gallbladder; and the anterior axillary line (AAL) at the point of the greatest craniocaudal extent. Splenic length was obtained pole to pole in a left sided oblique longitudinal view encompassing the splenic hilum. Renal volume was derived from a frontal approach using the ellipsoid formula: length × width × depth × 0.523 (Supplemental Fig. 1). Each measurement was obtained once. Weight, length, head circumference, and corrected gestational age were recorded on the day of the scan. Consecutive inclusion and a shared protocol with established interobserver reproducibility limited selection and measurement bias.

### Statistical analysis

All analyses were performed using GraphPad Prism 11.0.0 (GraphPad Software, Boston, MA, USA). Normality of continuous variables was assessed using the Shapiro–Wilk test. Normally distributed data are presented as mean ± SD, non-normally distributed data as median and interquartile range (IQR). No a priori sample size calculation was carried out. The study was exploratory, and as the research question had not been investigated previously, no empirical effect size estimate was available for a power analysis. The sample size was determined by the number of children who met the inclusion criteria during the recruitment period, reflecting the limited availability of this patient population at a single center [13].

To assess comparability with the reference AGA cohort, gestational age at birth and postnatal age, weight, length, and corrected gestational age at examination were compared between the two cohorts. An independent-samples t-test was used where both groups were normally distributed, and the Mann–Whitney U test (MWU) otherwise. Organ measurements within the SGA cohort were compared between male and female infants. Normality was assessed separately within each sex for every organ parameter; an independent-samples t-test was used where both groups were normally distributed, and the MWU was used otherwise. Associations between organ measurements and body length, body weight, and corrected gestational age at examination were assessed using Pearson's correlation coefficient (*r*) for normally distributed variables and Spearman's rank correlation coefficient (ρ) otherwise.

Organ dimensions were compared against previously published RIs for liver length, spleen length, and renal volume in eutrophic preterm infants below 40 cm body length [8]. For each organ parameter, the proportion of infants with a measured value outside the published RI was determined and compared against the 5% expected by definition of a 95% RI. A one-sample binomial test was used to assess whether the observed proportion was significantly higher than expected. Exact (Clopper-Pearson) 95% confidence intervals (CI) were calculated. Infants with a liver or spleen measurement outside the RI were additionally compared with the remaining infants regarding gestational age at birth, corrected gestational age at examination and somatic parameters at birth and examination, using the MWU given the small subgroup sizes.

To evaluate whether the cohort deviated systematically from the RI, z-scores were additionally calculated for each infant and organ parameter as (ln[measured value]—μ)/σ, where μ and σ are the published mean and standard deviation of the log-transformed measurements in the reference cohort [8]. Mean z-scores per organ were tested against zero using a one-sample t-test.

Because organ size correlates most strongly with body weight, a post hoc analysis accounted for weight. In the reference cohort (*n* = 57 for liver; *n* = 56 for spleen length and kidney volumes because of missing values), the log-transformed measurement was regressed on weight at examination. Weight-adjusted z-scores of SGA infants (observed minus predicted log-measurement, divided by the residual standard deviation) were tested against zero (one-sample t-test); an absolute z-score above 1.96 was regarded as outside the weight-specific limits. In the pooled cohorts, the difference between SGA and reference infants at equal weight is reported as percentage difference with 95% confidence interval. Sensitivity analyses used log-transformed weight, only SGA infants within the weight and age range of the reference cohort (*n* = 17), corrected gestational age as an additional covariate, and a cohort-by-weight interaction. Proportions outside the RI were also compared within weight tertiles of the reference cohort (Fisher’s exact test). These analyses were exploratory and not adjusted for multiple testing (Supplemental Tables 4 to 6). Statistical significance was defined as *p* < 0.05.

## Results

**Fig. 1:**
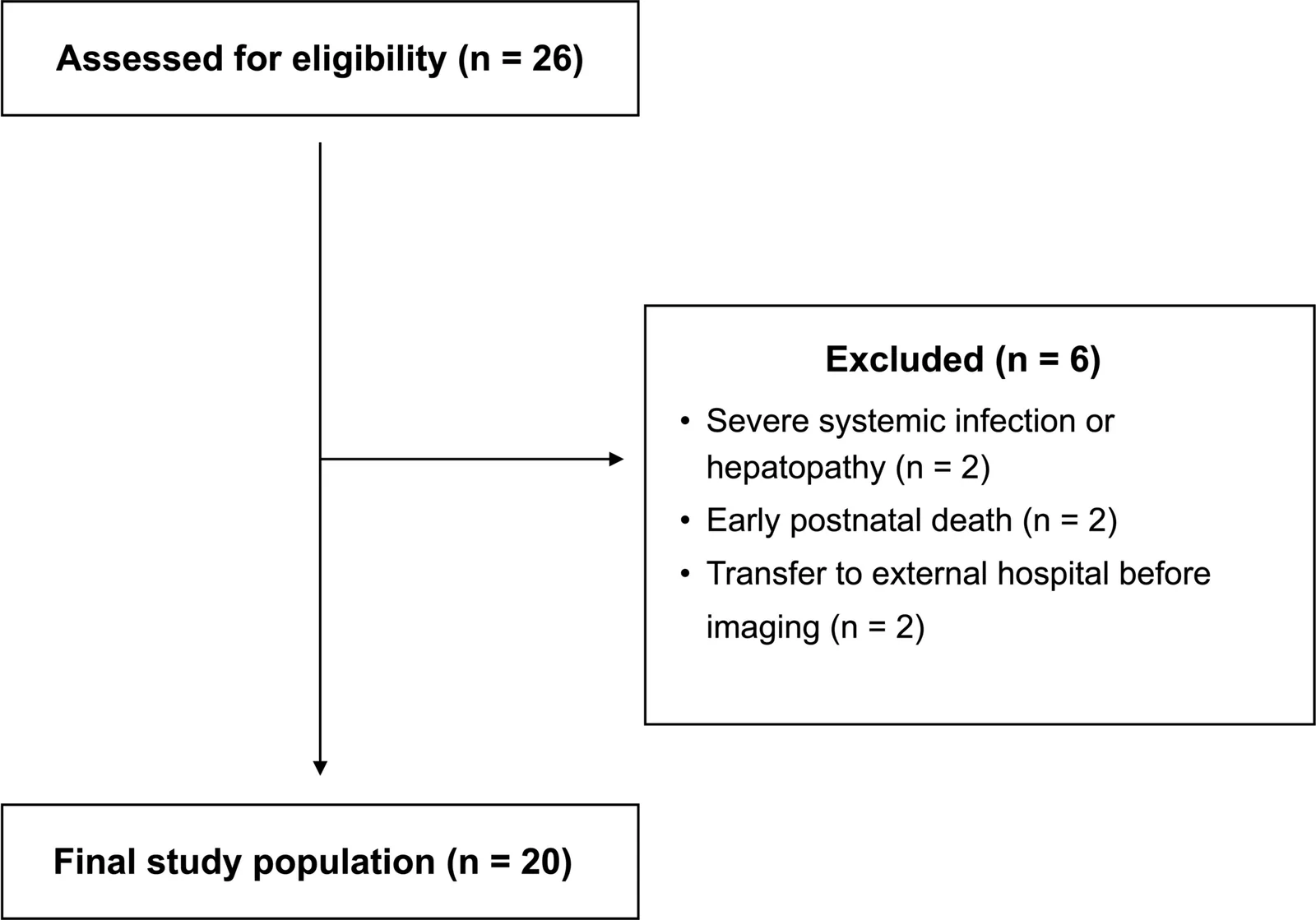
Participant flow diagram

Of 26 infants assessed for eligibility, two died and two were transferred to an external hospital before imaging, one was excluded for sepsis, and one for unclear hepatopathy. The final cohort comprised 20 infants (Fig. 1) and included 11 male and 9 female infants. Mean gestational age at birth was 29 ^1^/_7_ ± 3 ^1^/_7_ weeks. Median birth weight was 695 g (IQR 560—1186), corresponding to a median birth weight percentile of 4 (IQR 1—7). Mean birth length was 33.38 ± 3.09 cm. The first ultrasound examination was performed at a median postnatal age of 15.5 days (IQR 12.25—21.75). Renal volume was assessable in 18 infants as two were excluded due to a duplex kidney and a horseshoe kidney, with liver and spleen data retained for both (Table 1). No significant sex differences were observed for any organ measurement (t-test or MWU as appropriate, *p* = 0.208—0.837; Supplemental Table 1). Compared with the reference cohort, the SGA cohort had a significantly higher gestational age at birth and a higher corrected gestational age at examination. Postnatal age, weight, and length at examination did not differ significantly between cohorts (Table 2). One SGA infant exceeded the weight range of the reference cohort (1810 g vs. 1600 g), and two exceeded its range of postnatal age at examination (94 and 40 days vs. 32 days).

**Table 1:** Clinical, demographic, and sonographic characteristics of the study population

Characteristics	Value^*^
Number of infants	20
Sex	Female: 9 (45%) Male: 11 (55%)
Gestational age (p.m., weeks)	29 ^1^/_7_ ± 3 ^1^/_7_ Range: 23 ^2^/_7_—35 ^1^/_7_
Birth weight (g)	695 (560—1186) Range: 420—1450
Birth weight percentile	4 (1—7) Range: 1—9
Birth length (cm)	33.38 ± 3.09 Range: 28.50—39
Birth length percentile	3 (1—16) Range: 1—40
Birth head circumference (cm)	24.48 ± 2.91 Range: 20—29.5
Birth head circumference percentile	7 (1—9) Range: 1—40
Body surface area (Mosteller, m^2^)	0.08 (0.07—0.11) Range: 0.06—0.12
Age at first examination (days)	15.5 (12.25—21.75) Range: 1—94
Weight at first examination (g)	1011 ± 350 Range: 570—1810
Length at first examination (cm)	34.95 ± 3.17 Range: 30—39
Head circumference at first examination (cm)	25.91 ± 2.91 Range: 22—31.5
Liver length in MSL (cm)	3.06 ± 0.45 Range: 2.2—3.9
Liver length in MCL (cm)	3.55 ± 0.55 Range: 2.7—4.6
Liver length in AAL (cm)	3.64 ± 0.61 Range: 2.8—5.2
Spleen length (cm)	2.75 (2.50—3.25) Range: 2.3—4.3
Right kidney volume (ml), *n* = 18	5 (4—6) Range: 2—13
Left kidney volume (ml), *n* = 18	5 (4—6.75) Range: 3—10
Portal vein peak velocity (cm/s)	16.90 ± 4.32 Range: 8—24
Patent ductus venosus (n)	13 (65%)
AST at examination (± 7 days; U/l)	30.25 ± 8.70 Range: 12—45
ALT at examination (± 7 days; U/l)	10.80 ± 4.84 Range: 4—25
Creatinine at examination (± 7 days; µmol/l)	39.60 ± 11.30 Range: 24—63

*Non-normally distributed data: median (interquartile range); normally distributed: mean ± SD. Ranges are given as minimum—maximum. Kidney volume was assessed in 18 infants (two were excluded: duplex kidney, horseshoe kidney). *AAL* anterior axillary line, *MCL* midclavicular line, *MSL* midsternal line, *SGA* small for gestational age

**Table 2:** Comparison of gestational age and somatic parameters between the SGA cohort and the previously described AGA reference cohort

Parameter	SGA cohort (*n* = 20)	Reference cohort (*n* = 57)	Test	*P* value
Gestational age at birth (weeks)	29 ^1^/_7_ ± 3 ^1^/_7_ Range: 23 ^2^/_7_—35 ^1^/_7_	27 ^1^/_7_ ± 2 ^0^/_7_ Range: 23 ^4^/_7_—31 ^5^/_7_	t-test	0.001
Postnatal age at examination (days)	15.50 (12.25—21.75) Range: 1—94	14.00 (6.00—16.00) Range: 2—32	MWU	0.146
Weight at examination (g)	1011 ± 350 Range: 570—1810	1020 ± 250 Range: 560—1600	t-test	0.933
Length at examination (cm)	34.95 ± 3.17 Range: 30—39	36.00 (35.00—39.00) Range: 30—39.5	MWU	0.159
Corrected gestational age at examination (weeks)	32 ^0^/_7_ ± 2 ^4^/_7_ Range: 28 ^1^/_7_—36 ^6^/_7_	29 ^0^/_7_ ± 1 ^6^/_7_ Range: 25 ^2^/_7_—33 ^1^/_7_	t-test	< 0.001

*Non-normally distributed data: median (interquartile range); normally distributed: mean ± standard deviation. Reference cohort values for gestational age at birth, weight, and length at examination are taken from the previously published cohort description (Ref. 8). An independent-samples t-test was used where both groups were normally distributed; the Mann–Whitney U test (MWU) was used otherwise. *p* < 0.05 was considered statistically significant

*AGA* appropriate for gestational age, *p.m.* post menstruationem, *SGA* small for gestational age

### Organ dimensions relative to published reference intervals

**Fig. 2:**
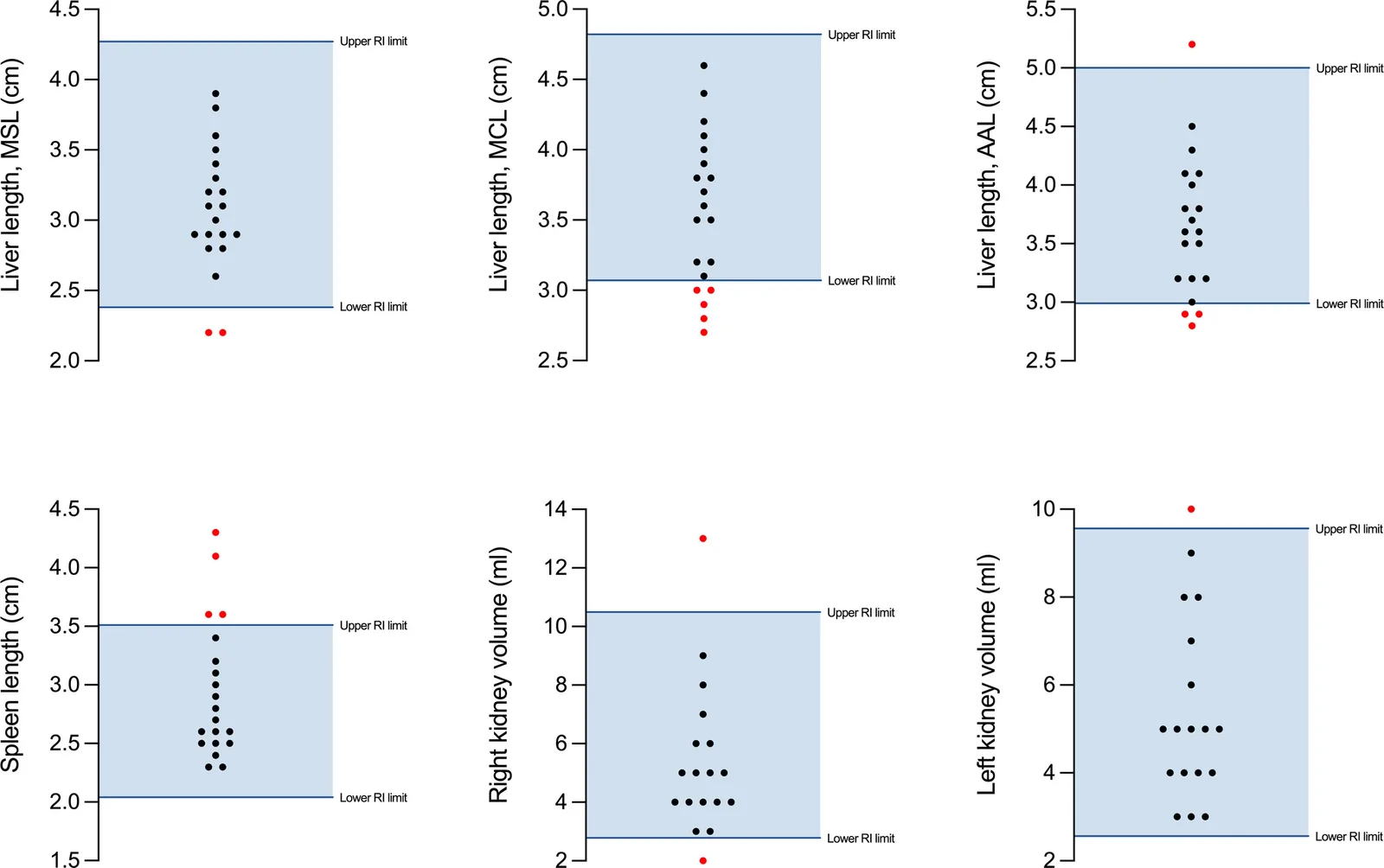
Individual liver, spleen, and kidney measurements in SGA infants relative to the AGA reference interval (blue shaded band). Liver length (MSL, MCL, AAL) and spleen length: *n* = 20; right and left kidney volume: *n* = 18 (two infants excluded for duplex kidney and horseshoe kidney). Red dots: outside reference interval. AAL: anterior axillary line, AGA: appropriate for gestational age, MCL: midclavicular line, MSL: midsternal line, RI: reference interval, SGA: small for gestational age

**Fig. 3:**
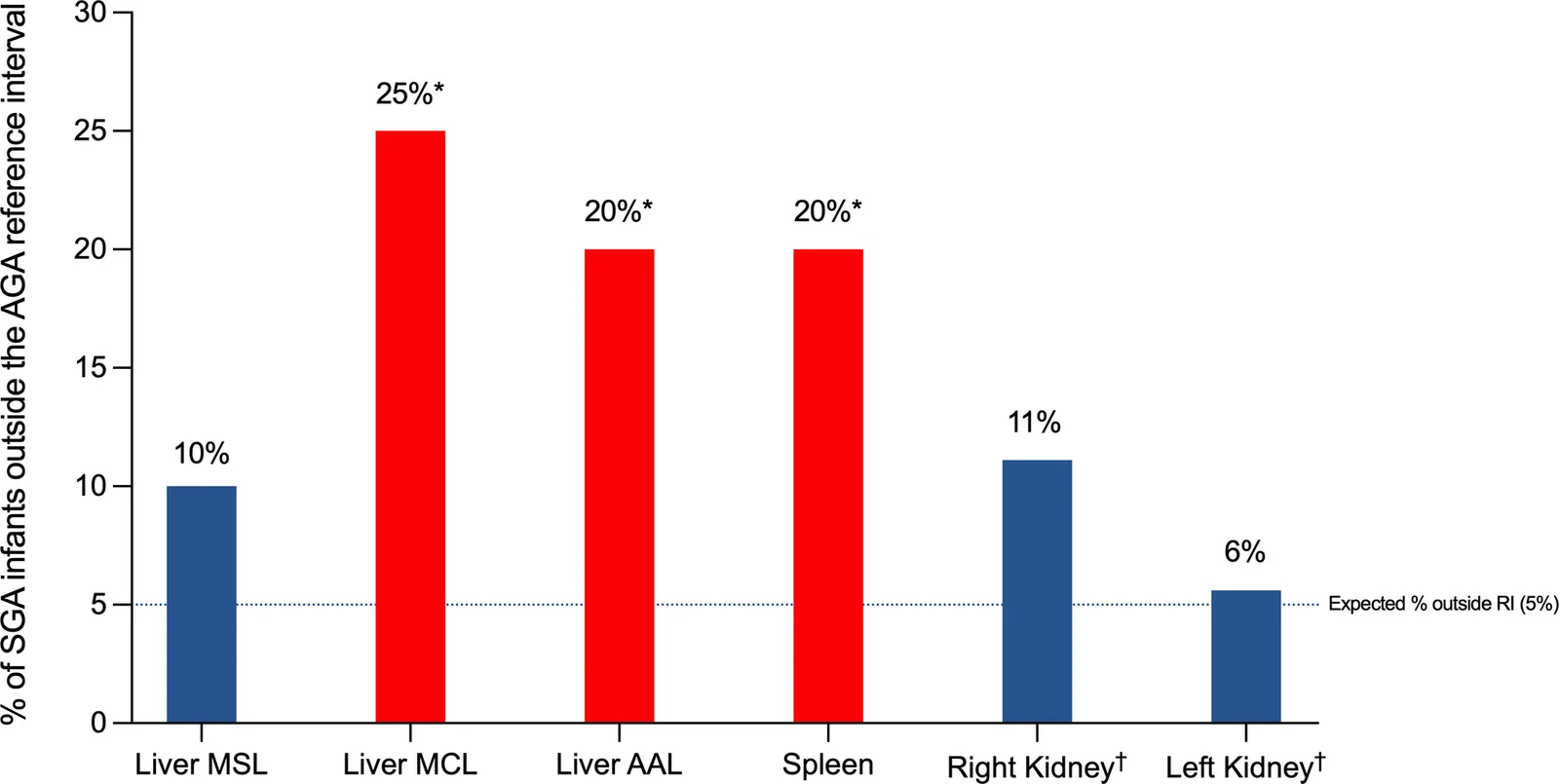
Proportion of SGA infants outside the AGA reference interval (RI), by organ. Dashed line: 5% expected. *p < 0.05. ^†^Kidney volume was assessed in 18 infants (two were excluded: duplex kidney, horseshoe kidney). AAL: anterior axillary line, AGA: appropriate for gestational age, MCL: midclavicular line, MSL: midsternal line, SGA: small for gestational age

The proportion of infants falling outside the published RI was compared against the 5% expected by definition of a 95% RI. This proportion was significantly elevated for liver length in the MCL (5 of 20 infants, 25.0%, 95% CI 8.7—49.1, *p* = 0.003), liver length in the AAL (4 of 20 infants, 20.0%, 95% CI 5.7—43.7, *p* = 0.016), and spleen length (4 of 20 infants, 20.0%, 95% CI 5.7—43.7, *p* = 0.016). In each of these three parameters, two outlying values lay within 1 mm of the reference limit. For all other parameters, the observed proportion did not differ significantly from 5% (Figs. 2 and 3). Of the 11 outlying liver values, 10 fell below the lower reference limit and one above the upper (AAL, 5.2 cm); all outlying spleen values exceeded the upper limit. Infants with a liver measurement below the RI had a significantly lower weight and length at examination than the remaining infants, despite a comparable gestational age at birth. Infants with a spleen measurement above the RI had a significantly higher corrected gestational age and were significantly heavier and longer at examination. All other comparisons are shown in Supplemental Table 2.

**Fig. 4:**
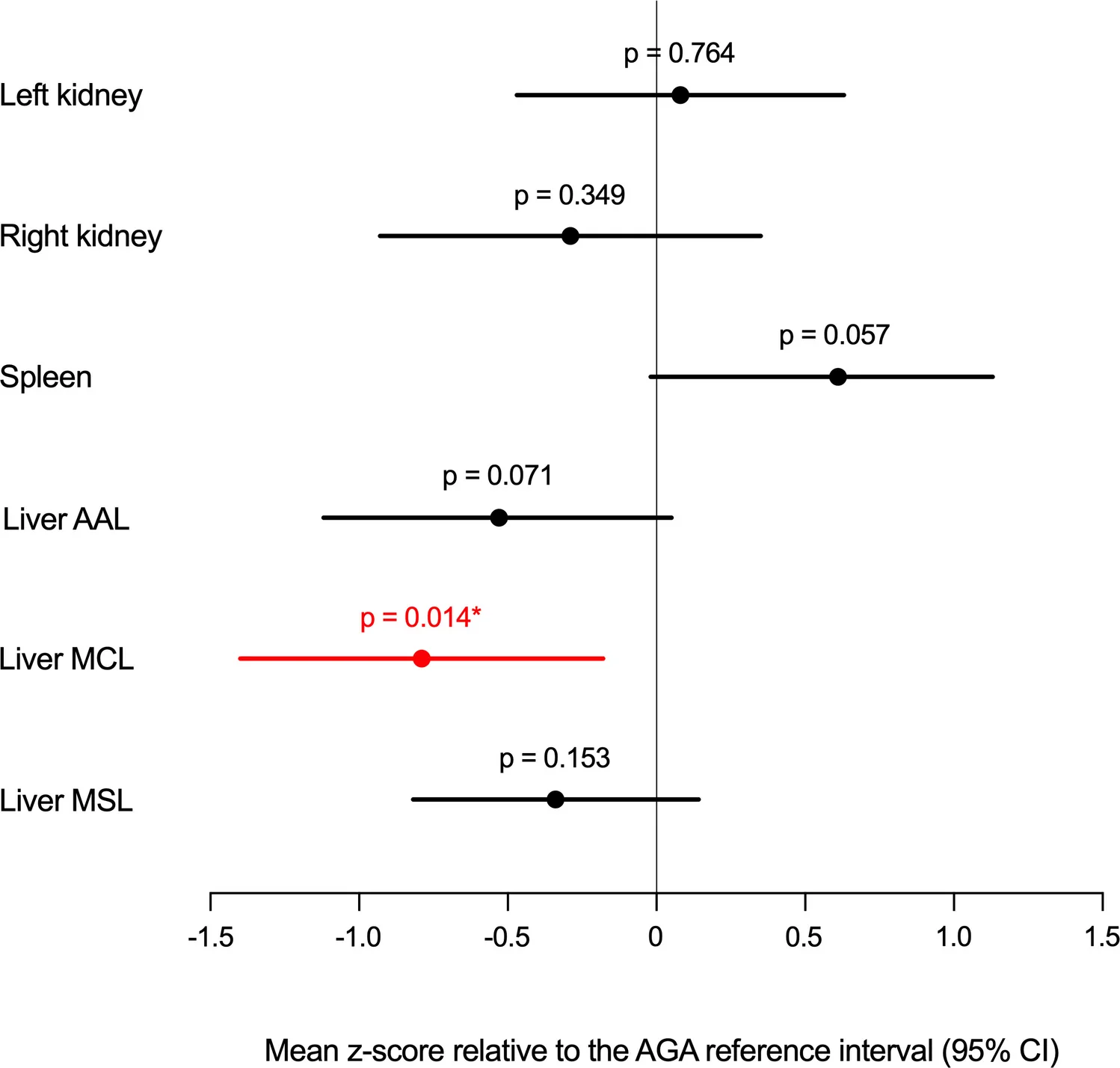
Mean z-score of the SGA cohort relative to the AGA reference interval, with 95% confidence interval (CI). *p < 0.05. AAL: anterior axillary line, AGA: appropriate for gestational age, MCL: midclavicular line, MSL: midsternal line, SGA: small for gestational age

Z-scores were calculated for each infant and organ parameter relative to the published RIs, and the mean z-score per organ was tested against zero. The mean z-score for liver length in the MCL was significantly below zero (mean z = − 0.79, *p* = 0.014); no other parameter deviated significantly from zero (Fig. 4).

Organ measurements correlated most strongly with body weight, followed by body length and corrected gestational age at examination. Correlations were strongest for renal volume, followed by spleen length. Liver length showed somewhat weaker associations overall, particularly with corrected gestational age (Supplemental Table 3).

### Additional analysis accounting for body weight (post hoc)

Because organ size correlated most strongly with body weight, each measurement was also compared with the value expected for the infant’s weight in the reference cohort. At equal weight, SGA infants had a shorter liver in the MCL (− 8.6%, 95% CI − 13.5 to − 3.3) and AAL (− 6.7%, − 12.2 to − 0.9) and a longer spleen (+ 8.6%, 2.1 to 15.6), whereas liver length in the MSL and both kidney volumes did not differ; weight-adjusted z-scores agreed (Supplemental Table 4). These differences persisted in sensitivity analyses, including restriction to the weight and age range of the reference cohort, where right kidney volume was also lower (*p* = 0.046), but not when corrected gestational age was added (Supplemental Table 5). Within the lightest weight tertile, liver length in the MCL was below the RI in 5 of 10 SGA and 1 of 20 reference infants (*p* = 0.009), and within the heaviest tertile, spleen length was above the RI in 4 of 7 SGA and none of 19 reference infants (*p* = 0.002); the other 16 comparisons were not significant (Supplemental Table 6).

## Discussion

The proportion of measurements outside the AGA RIs was significantly raised for liver length in the MCL, liver length in the AAL, and spleen length. These findings suggest that RIs derived from an AGA cohort should be applied with caution in infants born SGA.

The lower liver measurements are consistent with known alterations in fetal hepatic perfusion associated with placental insufficiency, including increased ductus venosus shunting and redistribution of blood flow away from the liver, particularly the right lobe. This redistribution intensifies with increasing circulatory compromise and may impair hepatic growth [14, 15]. Notably, reduced liver size does not appear to affect all SGA fetuses uniformly: in a study measuring liver length shortly before delivery, only 18% of SGA fetuses had a liver length below the 10th percentile, and this subgroup was characterized by more severe growth restriction overall [16]. In our cohort, likewise, only a subset of infants fell outside the RI. These infants had a significantly lower birth weight percentile despite a comparable gestational age at birth, suggesting a more pronounced degree of smallness rather than greater prematurity, and were also significantly lighter at examination than the remaining infants.

In contrast, four SGA infants had splenic dimensions above the upper limit of the AGA RI. Abuhamad et al. found a reduced splenic artery resistance index in the majority of SGA fetuses studied, attributed to erythropoietin-driven vasodilation in response to chronic hypoxia [17]. A comparable combination of reduced splenic resistance and splenic enlargement has been described in fetal anemia, where increased erythropoiesis drives both changes [18], supporting the plausibility of a similar mechanism in growth-restricted fetuses.

The affected infants were significantly heavier, longer, and had a higher corrected gestational age at examination than the remaining infants; as spleen length correlated significantly with these parameters, larger body size and higher maturity may have contributed to values outside the reference interval. However, spleen length remained larger in SGA infants at equal body weight (Supplemental Table 4), so body size alone does not explain the finding. As neither Doppler nor hematologic assessment was performed, the proposed mechanism cannot be confirmed from our data.

Renal volume showed no significant deviation from the RI, in either the proportion outside the RI or the mean z-score, despite its strongest correlations with somatic parameters among all organs assessed. This contrasts with a previous case–control study reporting significant differences in renal length and transverse diameter between preterm SGA and AGA infants by the seventh day of life [7]. This discrepancy may reflect the renal parameter assessed (volume versus linear dimensions), the timing of assessment, or a genuine absence of renal involvement. With only 18 infants assessed, a true effect missed because of limited sample size cannot be excluded.

The comparability analysis supports the interpretation that these deviations reflect SGA status rather than current body size. Weight, length, and postnatal age at examination did not differ significantly between the cohorts, whereas gestational age at birth and corrected gestational age at examination were higher in the SGA cohort, consistent with SGA infants reaching a given body length at a more advanced gestational age. Because organ size correlates most strongly with body weight, we also compared each organ with the size expected for the infant’s weight (post hoc analysis). The deviations for liver length in the MCL and AAL and for spleen length persisted at equal weight, whereas kidney volumes remained unremarkable (Supplemental Tables 4 and 5). They were also organ-specific: among the lightest infants the liver was small but spleen and kidneys were not, and among the heaviest infants the spleen was large but the liver was not (Supplemental Table 6). This pattern argues against a uniform effect of body size, although the subgroups are small. Weight-specific limits would not have removed the deviations either: even with weight accounted for, 4 of 20 infants for liver length in the MCL and 5 of 20 for spleen length fell outside the limits (Supplemental Table 4).

An organ size that falls outside the expected range can have diagnostic consequences. Liver enlargement may be caused by infection, congestive heart failure or metabolic liver disease [19–21]; splenic enlargement may indicate infection or portal hypertension [22, 23]; and renal enlargement may suggest renal dysplasia, a duplex system or renal vein thrombosis [24–26]. A small organ raises the question of hypoplasia. Such interpretations require RIs derived from comparable infants. Applying AGA RIs to SGA infants can cause misclassification in either direction. If organ size is shifted downwards in SGA infants, a diseased, enlarged organ may still fall within the AGA RI and be considered normal; conversely, a small but physiologically normal organ may fall below the AGA RI and be misread as hypoplastic, prompting unnecessary investigation. Given the increased risk of mortality and major morbidity in SGA infants (2), both missed diagnoses and unnecessary investigations carry more weight.

## Limitations

The sample size was small and determined by the availability of eligible infants during the recruitment period; exclusion criteria further reduced the cohort from 26 to 20 infants. This limited the power to detect deviations of moderate magnitude. Each significant proportion rested on 4 or 5 infants, several values lay within 1 mm of the reference limit, and even the significant z-score finding for liver length in the MCL should be interpreted with caution given the single-center, exploratory design. The weight-adjusted analyses were post hoc, based on a reference cohort of 56 to 57 infants, and not adjusted for multiple testing; they are hypothesis-generating. Weight and corrected gestational age were highly correlated (Supplemental Table 5), and 6 of 20 SGA infants exceeded the corrected gestational age of the reference cohort. The effects of growth restriction and maturity could therefore not be separated, and the model including corrected gestational age partly rests on extrapolation, so its non-significant result does not exclude an effect. Three SGA infants exceeded the weight or age range of the reference cohort. The cross-sectional design does not capture how organ size relative to the reference evolves during or after the neonatal stay, and the single-center setting may limit generalizability to other patient populations or ultrasound practices. Two infants were excluded from the renal volume analysis because of isolated structural anomalies unrelated to growth restriction, further reducing the sample size for this parameter. SGA was defined by birth weight percentile; fetal growth restriction could not be classified without fetal Doppler or growth trajectories. The mechanisms proposed for the hepatic and splenic findings apply only to growth-restricted fetuses and are speculative, as this study included neither fetal nor postnatal Doppler assessment nor hematologic parameters that could support or refute them.

## Conclusions

In conclusion, abdominal organ dimensions in SGA preterm infants frequently fall outside AGA-derived reference intervals, suggesting that these intervals cannot be reliably applied to this population. The deviations were not explained by body weight alone, although growth restriction and maturity could not be separated in this cohort. Prospective studies with larger sample sizes establishing dedicated reference values for SGA infants are warranted.

## Supplementary Information

Below is the link to the electronic supplementary material.

Fig. 5 (PNG 6.31 MB)

Supplementary file1 (TIFF 8628 KB)

Supplementary file2 (DOCX 15 KB)

Supplementary file3 (DOCX 34.5 KB)

Supplementary file4 (DOCX 38 KB)

Supplementary file5 (DOCX 47 KB)

## Data Availability

The datasets generated and analyzed during this study are not publicly available due to patient confidentiality regulations but are available from the corresponding author upon reasonable request.

## Abbreviations

AAL: Anterior axillary line
AGA: Appropriate for gestational age
CI: Confidence interval
DEGUM: German Society for Ultrasound in Medicine
FGR: Fetal growth restriction
ICC: Intraclass correlation coefficient
IQR: Interquartile range
MCL: Midclavicular line
MSL: Midsternal line
MWU: Mann-Whitney U test
NICU: Neonatal intensive care unit
RI: Reference interval
SD: Standard deviation
SGA: Small for gestational age
