# Supplementary material for: Organ size in small infants (The OSSI Study): are sonographic reference intervals applicable in preterm infants small for gestational age?
Source: Eur J Pediatr. 2026 Oct 28;185(11):898. doi: 10.1007/s00431-026-07580-4 (PMC13719713; doi:10.1007/s00431-026-07580-4)
Supplement: Supplementary file 1 [file 431_2026_7580_MOESM2_ESM.docx]

**Supplemental Table 1** Comparison of organ measurements between male and female SGA preterm infants.

*Non-normally distributed data: median (interquartile range); normally distributed: mean ± standard deviation. Normality was assessed separately for male and female infants. An independent-samples t-test was used where both groups were normally distributed; the Mann-Whitney U test (MWU) was used otherwise. Kidney volume was assessed in 18 infants (two were excluded: duplex kidney, horseshoe kidney). p < 0.05 was considered statistically significant. AAL: anterior axillary line, CI: confidence interval, MCL: midclavicular line, MSL: midsternal line, SGA: small for gestational age.

| Organ | n (male) | **Value (male)** | **n (female)** | **Value (female)** | **Mean difference (m - f)** | **95% CI of the difference** | **Test** | **p-value** |
| --- | --- | --- | --- | --- | --- | --- | --- | --- |
| Liver length in MSL (cm) | 11 | 3.05 ± 0.47 | 9 | 3.09 ± 0.45 | -0.04 | -0.48 - 0.39 | t-test | 0.837 |
| Liver length in MCL (cm) | 11 | 3.47 ± 0.63 | 9 | 3.64 ± 0.46 | -0.17 | -0.70 - 0.36 | t-test | 0.504 |
| Liver length in AAL (cm) | 11 | 3.53 ± 0.55 | 9 | 3.79 ± 0.68 | -0.26 | -0.84 - 0.31 | t-test | 0.353 |
| Spleen length (cm) | 11 | 3.00 (2.65 - 3.30) | 9 | 2.60 (2.50 - 2.80) | n.a. | n.a. | MWU | 0.208 |
| Right kidney volume (ml), n = 18 | 10 | 5.00 ± 1.56 | 8 | 5.88 ± 3.56 | -0.88 | -3.52 - 1.77 | t-test | 0.494 |
| Left kidney volume (ml), n = 18 | 10 | 5.30 ± 1.64 | 8 | 5.62 ± 2.72 | -0.32 | -2.52 - 1.87 | t-test | 0.757 |

**a**

**c**
