# Supplementary material for: Organ size in small infants (The OSSI Study): are sonographic reference intervals applicable in preterm infants small for gestational age?
Source: Eur J Pediatr. 2026 Oct 28;185(11):898. doi: 10.1007/s00431-026-07580-4 (PMC13719713; doi:10.1007/s00431-026-07580-4)
Supplement: Supplementary file 2 [file 431_2026_7580_MOESM3_ESM.docx]

**Supplemental Table 2** Comparison of somatic characteristics between SGA infants with an organ measurement outside the AGA reference interval (RI) and the remaining SGA infants.

a) Liver length below the reference interval (any of three planes)

| Parameter | Liver length below RI (n = 5) | Liver within RI (n = 15) | **p value** |
| --- | --- | --- | --- |
| Gestational age at birth (weeks) | 28 ^3^/_7_ (27 ^6^/_7_ - 29 ^1^/_7_) | 29 ^4^/_7_ (27 ^1^/_7_ - 31 ^4^/_7_) | 0.727 |
| Birth weight percentile | 1 (1 - 1) | 5 (4 - 7) | 0.012 |
| Birth weight (g) | 530 (500 - 700) | 905 (603 - 1208) | 0.142 |
| Birth length percentile | 1 (1 - 1) | 5 (1 - 20) | 0.109 |
| Birth length (cm) | 31 (29 - 32) | 34 (32 - 37) | 0.035 |
| Birth head circumference percentile | 1 (1 - 1) | 8 (6 - 13) | 0.004 |
| Birth head circumference (cm) | 22 (22 - 22.5) | 26.5 (23 - 27) | 0.049 |
| Weight at examination (g) | 700 (660 - 765) | 1090 (850 - 1340) | 0.018 |
| Length at examination (cm) | 32 (31 - 33) | 36 (33.5 - 39) | 0.019 |
| Corrected gestational age at examination (weeks) | 30 ^6^/_7_ (30 ^3^/_7_ - 31 ^1^/_7_) | 31 ^6^/_7_ (30 ^0^/_7_ - 34 ^5^/_7_) | 0.432 |

**b)** Spleen length above the reference interval

| Parameter | Spleen length above RI (n = 4) | Spleen length within RI (n = 16) | **p value** |
| --- | --- | --- | --- |
| Gestational age at birth (weeks) | 31 ^1^/_7_ (28 ^6^/_7_ - 32 ^3^/_7_) | 28 ^2^/_7_ (27 ^2^/_7_ - 31 ^2^/_7_) | 0.508 |
| Birth weight percentile | 6.5 (4.5 - 7.5) | 4 (1 - 5.5) | 0.332 |
| Birth weight (g) | 1195 (998 - 1216) | 670 (560 - 952) | 0.385 |
| Birth length percentile | 2.5 (1 - 13) | 4.5 (1 - 16) | 0.882 |
| Birth length (cm) | 36 (34 - 37) | 32 (31 - 35) | 0.419 |
| Birth head circumference percentile | 8.5 (6 - 17) | 6 (1 - 9) | 0.383 |
| Birth head circumference (cm) | 27.5 (25 - 29) | 23.5 (22.5 - 27) | 0.343 |
| Weight at examination (g) | 1340 (1320 - 1390) | 790 (700 - 1030) | 0.023 |
| Length at examination (cm) | 39 (38.5 - 39) | 34 (32 - 36) | 0.011 |
| Corrected gestational age at examination (weeks) | 35 ^3^/_7_ (33 ^6^/_7_ - 36 ^4^/_7_) | 30 ^0^/_7_ (30 ^1^/_7_ - 31 ^6^/_7_) | 0.012 |

*All comparisons were performed using the Mann-Whitney U test; a formal assessment of normality was not considered reliable at this sample size. Data are presented as median (interquartile range). p < 0.05 was considered statistically significant. AGA: appropriate for gestational age, RI: reference interval, SGA: small for gestational age.

**a**

**c**
