# Supplementary material for: Organ size in small infants (The OSSI Study): are sonographic reference intervals applicable in preterm infants small for gestational age?
Source: Eur J Pediatr. 2026 Oct 28;185(11):898. doi: 10.1007/s00431-026-07580-4 (PMC13719713; doi:10.1007/s00431-026-07580-4)
Supplement: Supplementary file 3 [file 431_2026_7580_MOESM4_ESM.docx]

**Supplemental Table 3** Correlation coefficients, 95% confidence intervals (CI), and P values for associations between organ measurements and body weight (a), body length (b), and corrected gestational age (c) at examination.

**a)** Correlation with body weight at examination

**b)** Correlation with body length at examination

| Organ dimension | Method | ***r* / ρ** | **95% CI** | **P value** |
| --- | --- | --- | --- | --- |
| Liver length in MSL (cm) | Pearson | 0.453 | 0.013 - 0.746 | 0.045 |
| Liver length in MCL (cm) | Pearson | 0.462 | 0.025 - 0.751 | 0.040 |
| Liver length in AAL (cm) | Pearson | 0.468 | 0.032 - 0.754 | 0.038 |
| Spleen length (cm) | Spearman | 0.600 | 0.214 - 0.824 | 0.005 |
| Right kidney volume (ml) | Spearman | 0.612 | 0.203 - 0.839 | 0.007 |
| Left kidney volume (ml) | Spearman | 0.751 | 0.438 - 0.902 | <0.001 |

| Organ dimension | Method | ***r* / ρ** | **95% CI** | **P value** |
| --- | --- | --- | --- | --- |
| Liver length in MSL (cm) | Pearson | 0.517 | 0.097 - 0.781 | 0.020 |
| Liver length in MCL (cm) | Pearson | 0.466 | 0.029 - 0.753 | 0.039 |
| Liver length in AAL (cm) | Pearson | 0.566 | 0.164 - 0.806 | 0.009 |
| Spleen length (cm) | Spearman | 0.661 | 0.309 - 0.854 | 0.002 |
| Right kidney volume (ml) | Spearman | 0.710 | 0.363 - 0.884 | 0.001 |
| Left kidney volume (ml) | Spearman | 0.811 | 0.555 - 0.927 | <0.001 |

**c)** Correlation with corrected gestational age at examination

| Organ dimension | Method | ***r* / ρ** | **95% CI** | **P value** |
| --- | --- | --- | --- | --- |
| Liver length in MSL (cm) | Pearson | 0.212 | -0.254 - 0.598 | 0.369 |
| Liver length in MCL (cm) | Pearson | 0.232 | -0.234 - 0.612 | 0.324 |
| Liver length in AAL (cm) | Pearson | 0.460 | 0.022 - 0.750 | 0.041 |
| Spleen length (cm) | Spearman | 0.666 | 0.317 - 0.856 | 0.001 |
| Right kidney volume (ml) | Spearman | 0.560 | 0.126 - 0.814 | 0.016 |
| Left kidney volume (ml) | Spearman | 0.729 | 0.397 - 0.892 | <0.001 |

*Pearson's *r* or Spearman's ρ as appropriate. Kidney volume, n = 18; all others, n = 20. p < 0.05 was considered statistically significant. AAL: anterior axillary line, CI: confidence interval, MCL: midclavicular line, MSL: midsternal line.

**a**

**c**
