# Supplementary material for: Organ size in small infants (The OSSI Study): are sonographic reference intervals applicable in preterm infants small for gestational age?
Source: Eur J Pediatr. 2026 Oct 28;185(11):898. doi: 10.1007/s00431-026-07580-4 (PMC13719713; doi:10.1007/s00431-026-07580-4)
Supplement: Supplementary file 4 [file 431_2026_7580_MOESM5_ESM.docx]

**Supplemental Table 4** Comparison of SGA infants with the reference cohort at equal body weight (post hoc analysis).

| Organ | n SGA/reference | **Difference at equal weight, % (95% CI)** | **p-value** | **Weight-adjusted z-score, mean (95% CI)** | **p-value** | **Infants with \|z\| > 1.96, n/N (%)** | **p (vs. 5%)** |
| --- | --- | --- | --- | --- | --- | --- | --- |
| Liver length in MSL | 20/57 | -4.7 (-10.7 to 1.8) | 0.154 | -0.38 (-0.90 to 0.14) | 0.139 | 3/20 (15.0) | 0.075 |
| Liver length in MCL | 20/57 | -8.6 (-13.5 to -3.3) | 0.002 | -0.94 (-1.61 to -0.26) | 0.009 | 4/20 (20.0) | 0.016 |
| Liver length in AAL | 20/57 | -6.7 (-12.2 to -0.9) | 0.024 | -0.63 (-1.21 to -0.06) | 0.033 | 3/20 (15.0) | 0.075 |
| Spleen length | 20/56 | +8.6 (+2.1 to +15.6) | 0.010 | +0.76 (+0.13 to +1.39) | 0.020 | 5/20 (25.0) | 0.003 |
| Right kidney volume | 18/56 | -9.0 (-21.0 to +4.8) | 0.188 | -0.39 (-1.05 to +0.27) | 0.229 | 3/18 (16.7) | 0.058 |
| Left kidney volume | 18/56 | +3.0 (-9.0 to +16.5) | 0.639 | +0.13 (-0.39 to +0.65) | 0.607 | 0/18 (0) | 1.000 |

Difference at equal weight: percentage difference in organ size of SGA infants relative to reference infants of the same weight, from a linear model of the log-transformed measurement on weight at examination and cohort (exp(β) - 1). Weight-adjusted z-score: (observed log-measurement - value predicted from the regression of log-measurement on weight in the reference cohort) / residual SD of this regression; tested against zero (one-sample t-test). Proportion with |z| > 1.96 tested against 5% (one-sided exact binomial test). Reference cohort n = 56 for spleen length and kidney volumes (one missing value each). Heteroscedasticity-robust (HC3) standard errors led to the same conclusions. Exploratory analysis, p values not adjusted for multiple testing. AAL: anterior axillary line, CI: confidence interval, MCL: midclavicular line, MSL: midsternal line, SGA: small for gestational age.

**a**

**c**

**Supplemental Table 5** Sensitivity analyses of the difference between SGA and reference infants at equal body weight (post hoc analysis).

Cells show the percentage difference in organ size of SGA infants relative to reference infants at equal weight (exp(β) - 1), the 95% CI in parentheses, and the p value. Negative values: SGA infants smaller than reference infants of the same weight; positive values: larger. Model B: weight ≤ 1600 g and postnatal age ≤ 32 days; n = 17 SGA infants (kidney volumes: n = 15) and the full reference cohort. Model C: weight and corrected gestational age were strongly correlated (r = 0.81 in the reference cohort, r = 0.82 in the SGA cohort), and 6 of 20 SGA infants had a corrected gestational age above the maximum of the reference cohort (33 ^1^/_7_ weeks); estimates are therefore imprecise and partly extrapolated, and non-significance does not exclude an effect. Exploratory analysis, p values not adjusted for multiple testing. AAL: anterior axillary line, CI: confidence interval, MCL: midclavicular line, MSL: midsternal line, SGA: small for gestational age.

| Organ | Model A: log-transformed weight; difference, % (95% CI), p | **Model B: SGA infants within weight and age range of the reference cohort; difference, % (95% CI), p** | **Model C: log-transformed weight and corrected gestational age; difference, % (95% CI), p** | **Interaction cohort × log weight, p** |
| --- | --- | --- | --- | --- |
| Liver length in MSL | -4.0 (-10.1 to +2.6), 0.225 | -4.1 (-10.8 to +3.0), 0.246 | +6.6 (-3.5 to +17.6), 0.204 | 0.376 |
| Liver length in MCL | -8.0 (-13.0 to -2.7), 0.004 | -8.2 (-13.6 to -2.5), 0.006 | -3.1 (-11.2 to +5.6), 0.465 | 0.754 |
| Liver length in AAL | -6.1 (-11.7 to -0.2), 0.044 | -7.1 (-12.7 to -1.1), 0.022 | -2.5 (-11.4 to +7.2), 0.595 | 0.934 |
| Spleen length | +9.6 (+3.0 to +16.5), 0.004 | +10.3 (+3.4 to +17.6), 0.003 | +4.9 (-4.8 to +15.6), 0.325 | 0.845 |
| Right kidney volume | -6.4 (-18.3 to +7.3), 0.339 | -13.9 (-25.6 to -0.3), 0.046 | -1.5 (-20.3 to +21.8), 0.891 | 0.624 |
| Left kidney volume | +6.0 (-5.9 to +19.4), 0.336 | +1.5 (-11.2 to +16.0), 0.823 | +3.9 (-13.7 to +25.1), 0.684 | 0.378 |

**a**

**c**

**Supplemental Table 6** Proportion of infants outside the AGA reference interval, by weight tertile of the reference cohort (post hoc analysis).

| Weight tertile/parameter | Reference (AGA) infants with a value outside the RI, n/N | **SGA infants with a value outside the RI, n/N** | **p (Fisher's exact test, SGA vs. reference)** |
| --- | --- | --- | --- |
| **Lightest third (≤ 910 g)** |  |  |  |
| Liver length, MSL | 1/20 | 2/10 | 0.251 |
| Liver length, MCL | 1/20 | 5/10 | 0.009 |
| Liver length, AAL | 2/20 | 3/10 | 0.3 |
| Spleen length | 2/19 | 0/10 | 0.532 |
| Right kidney volume | 1/19 | 1/9 | 1 |
| Left kidney volume | 1/19 | 0/9 | 1 |
| **Middle third** |  |  |  |
| Liver length, MSL | 1/18 | 0/3 | 1 |
| Liver length, MCL | 0/18 | 0/3 | 1 |
| Liver length, AAL | 0/18 | 0/3 | 1 |
| Spleen length | 0/18 | 0/3 | 1 |
| Right kidney volume | 0/18 | 0/2 | 1 |
| Left kidney volume | 0/18 | 0/2 | 1 |
| **Heaviest third (≥ 1133 g)** |  |  |  |
| Liver length, MSL | 0/19 | 0/7 | 1 |
| Liver length, MCL | 0/19 | 0/7 | 1 |
| Liver length, AAL | 1/19 | 1/7 | 0.474 |
| Spleen length | 0/19 | 4/7 | 0.002 |
| Right kidney volume | 0/19 | 1/7 | 0.269 |
| Left kidney volume | 0/19 | 1/7 | 0.269 |

Each row compares, within one weight tertile, the proportion of reference (AGA) infants with the proportion of SGA infants whose measurement lay outside the reference interval (RI), below or above. Example: 5/10 means that 5 of 10 SGA infants in this weight tertile had a value outside the RI. In the lightest third, all SGA values outside the RI were below the lower limit; in the heaviest third, all were above the upper limit. Tertile limits were taken from the reference cohort (n = 57): lightest third ≤ 910 g (reference n = 20, SGA n = 10), middle third (reference n = 18, SGA n = 3), heaviest third ≥ 1133 g (reference n = 19, SGA n = 7). N is smaller for spleen length and kidney volumes in the reference cohort (one missing value each) and for kidney volumes in the SGA cohort (two infants excluded). Fisher's exact test, two-sided. Exploratory analysis (18 comparisons), p values not adjusted for multiple testing. AAL: anterior axillary line, AGA: appropriate for gestational age, MCL: midclavicular line, MSL: midsternal line,, RI: reference interval, SGA: small for gestational age.

**a**

**c**
